# Extracellular Matrix in Plants and Animals: Hooks and Locks for Viruses

**DOI:** 10.3389/fmicb.2017.01760

**Published:** 2017-09-12

**Authors:** Livia Stavolone, Vincenzo Lionetti

**Affiliations:** ^1^Istituto per la Protezione Sostenibile delle Piante, Consiglio Nazionale delle Ricerche Bari, Italy; ^2^International Institute of Tropical Agriculture Ibadan, Nigeria; ^3^Dipartimento di Biologia e Biotecnologie “C. Darwin”, “Sapienza” Università di Roma Rome, Italy

**Keywords:** extracellular matrix, pectin, cell to cell movement, plant and animal viruses, cell wall

## Abstract

The extracellular matrix (ECM) of animal and plants cells plays important roles in viral diseases. While in animal cells extracellular matrix components can be exploited by viruses for recognition, attachment and entry, the plant cell wall acts as a physical barrier to viral entry and adds a higher level of difficulty to intercellular movement of viruses. Interestingly, both in plant and animal systems, ECM can be strongly remodeled during virus infection, and the understanding of remodeling mechanisms and molecular players offers new perspectives for therapeutic intervention. This review focuses on the different roles played by the ECM in plant and animal hosts during virus infection with special emphasis on the similarities and differences. Possible biotechnological applications aimed at improving viral resistance are discussed.

## Introduction

The collection of extracellular molecules secreted by animal and plant cells is named Extracellular Matrix (ECM). ECM is generally composed of well-organized networks of polysaccharides and proteins, which play important functions in different tissues. It supports the cells in a tissue and regulates intercellular adhesion and communication. ECM serves as physical scaffold to the cell but it is also a dynamic structure remodeled by physiological cell conditions including homeostasis, survival, growth, migration and differentiation, as well as in response to diseases (Bellincampi et al., 2014; Bonnans et al., 2014; Humphrey et al., 2014). With the exception of animal and protozoal, the majority of cell types are covered by a cell wall (CW), a complex network of proteins and carbohydrates, in which phenolic compounds can also be deposited during particular physiological processes (Keegstra, 2010; Bellincampi et al., 2014). The name CW describes the characteristics of rigidity, support, and actual shape conferred by this particular ECM to plant cells (Guerriero et al., 2014). Besides the structural functions, plant CWs play critical physiological roles, among which build turgor pressure, control intercellular communication and defense response against pests and pathogens (Lionetti and Metraux, 2014; Lionetti et al., 2014a, 2015; Whitehill et al., 2016). More recently, strong evidence depicts the plant CW as a dynamic structure, largely remodeled to solve new physiological functions (Ebine and Ueda, 2015; Lionetti et al., 2017).

Viruses are obligate intracellular parasites that do not possess the molecular machinery to replicate without a host. They need to enter host living cells and get in contact with the cytoplasm (Dimitrov, 2004). The earliest and most important stage of virus infection is cell entry and the consequent transfer of viral genetic material (Smith and Helenius, 2004; Alsteens et al., 2017). After replication, viruses can move directly between adjacent cells and invade the host by mechanisms of spread that may strongly depend on the particular way the virus enter/exit the host cell. Plant CW represents a physical barrier to viral entry and adds a higher level of difficulty to intercellular movement of viruses (Lionetti et al., 2012; Knox and Benitez-Alfonso, 2014). A contrasting situation applies to ECM of animal cells whose components can act as viral receptors favoring viral recognition, attachment and entry into the cell.

## The Intricate Networks of ECMs in Plant and Animal Cells

Important constituents of the animal ECMs are proteoglycans (PGs) formed by a core protein onto which one or more glycosaminoglycans (GAGs) chains are covalently linked (Frantz et al., 2010; Kular et al., 2014; Theocharis et al., 2016). ECMs are also enriched in proteins such as collagens (the main structural protein in connective tissue), elastin, fibronectin, laminins and glycoproteins. GAGs are long and negatively charged heteropolysaccharides characterized by disaccharide repetitions of *N*-acetylated hexosamines and D-/L-hexuronic acid, which are substituted with sulfate groups at various positions. The main GAGs are the galactosaminoglycans chondroitin sulfate (CS) and dermatan sulfate (DS), and the glycosaminoglycans keratan sulfate (KS), Heparin (Hep), and Heparan sulfate (HS). Hyaluronan (HA) is also an important constituent of EMCs. Unique among GAGs, HA is biosynthesized at the cell membrane rather than at the Golgi apparatus, is non-sulfated and not linked to proteins. Cells embedded into ECMs interact with this macromolecular network through their surface receptors, such as integrins, discoidin domain receptors (DDRs), cell surface PGs, and the HA receptor CD44 (Bosman and Stamenkovic, 2003). Different cell types synthesize and secrete matrix macromolecules under the control of multiple signals. Variations in the composition and structure of ECM, that can be endogenously mediated by proteinases, such as the Matrix Metallo Proteinases (MMPs), affect both the overall structure and biomechanical properties of the formed network, but also the signals transmitted to cells, thus modulating their responses (Bonnans et al., 2014).

Similar to the animal ECM, the plant CW is composed primarily of polysaccharides of which cellulose is the major component (Caffall and Mohnen, 2009; McFarlane et al., 2014). The CW is organized into paracrystalline structures (micro- and macrofibrils) embedded in a rich matrix of diverse polysaccharides, including hemicelluloses and pectins, structural glycoproteins and lignin in certain tissues (Zablackis et al., 1995). Hemicelluloses include xyloglucan containing a (1,4)-β-linked glucan backbone substituted with (1,6)-α-linked xylosyl residues or side chains of xylosyl, galactosyl, and fucosyl residues. Pectins are a complex group of polysaccharides composed of Homogalacturonan (HG), Rhamnogalacturonan I (RGI), Rhamnogalacturonan II (RGII), and Xylogalacturonan. HG, a linear polymer of (1,4)-α- linked Galacturonic Acid (GalA) residues, is the prevalent component of leaf CW pectins and is critical for tissue integrity, wall plasticity and cell adhesion (Lionetti et al., 2010, 2014a; Pogorelko et al., 2013). While cellulose is synthesized at the plasma membrane (PM) (McNamara et al., 2015; Maleki et al., 2016), the plant secretory pathway plays a functional role in CW biosynthesis of non-cellulosic polysaccharides, glycoproteins and PG, which are synthesized in the Golgi apparatus by glycosyltransferases (Kim and Brandizzi, 2016; Temple et al., 2016).

## ECM and CW: Interactor and Barrier for Viral Entry

**FIGURE 1:**
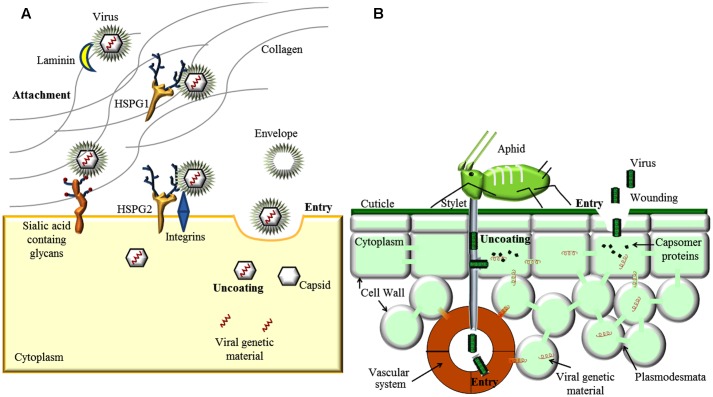
Involvement of Extracellular matrix and Cell Wall (CW) in virus entry. **(A)** In animal virus entry, the virus can bind to extracellular matrix (ECM) receptors, like Laminin, Heparan Sulfate Proteo Glycan 1 (HSPG 1) and integrins. Viruses can interact with secondary binding sites (HSPG 2 or sialic acid-containing glycans) present on the cell surface. Interaction with cell surface receptor can induce conformational triggering endocytosis. **(B)** Plant viruses can enter host cells and get in contact with the cytoplasm only via feeding of invertebrate vectors, e.g., aphids, or trough mechanical wounding involving partial destruction of the CW. Once inside the cell cytoplasm, both animal and plant viruses are uncoated and replicated following similar routes.

To initiate infection, animal viruses face the extracellular matrix of animal cells before traversing the host-cell PM. ECM represents a formidable barrier but different viruses evolved specific strategies to overcome and even exploit it for cell entry. Viral entry starts with attachment to cell-surface receptors and ends with the transfer of the viral genome to the cytoplasm (Dimitrov, 2004). After recognition and binding of cell surface receptors, which can be proteins, carbohydrates or lipids, viruses can enter cells via endocytosis. ECM appears to be involved in the attachment, the first steps of virus entry (**Figure 1A**). The majority of Papillomaviruses (PV) use HS as the primary attachment receptors (Sapp and Bienkowska-Haba, 2009; DiGiuseppe et al., 2017). The number and type of sulfation can influence virus attachments and infection (Knappe et al., 2007). The Laminin 5, a high-molecular weight protein of the extracellular matrix, shows high affinity to human papillomavirus type 11 (HPV11) virions and, in addition to HS, can mediate binding to ECM (Richards et al., 2014). HS and glycosphingolipids as well as carbohydrate-binding proteins like lectins, are thought to act as co-receptor molecules, which enhance the efficiency of entry of dengue virus, causing fever and hemorrhagic disorders in humans and non-human primates (Hidari and Suzuki, 2011). It is hypothesized that some hepatitis C virus (HCV) glycoproteins attach to lectins on the host cell surface (liver cells) for infection (Bartenschlager and Sparacio, 2007). The initial interaction of herpes simplex virus (HSV) is mediated via interactions with HS (Akhtar and Shukla, 2009). Also integrins, have been implicated as putative HSV and human immunodeficiency virus (HIV) receptors (Parry et al., 2005; Ding et al., 2015). Sialic acid-containing glycans are used by many viruses, like Influenza-, Parainfluenza-, Mumps-, Corona-, Noro-, Rotavirus, and DNA tumor viruses, as receptors for cell entry (Stencel-Baerenwald et al., 2014; Matrosovich et al., 2015).

In plant, the CW is an effective selective filter with an exclusion limit of approximately 60 kDa that allows diffusion of water, ions and signaling molecules but excludes virus particles (Tepfer and Taylor, 1981). Crossing the CW is a major challenge for viruses and such complex process is not yet fully understood. Viruses can enter host cells and get in contact with the cytoplasm only through mechanical wounding involving partial destruction of the CW and perforation of the PM, or via feeding of invertebrate vectors such as fungi, nematodes or insects (Hull, 2013) (**Figure 1B**). In addition, viruses can be vertically transmitted through seeds or by vegetative propagation (Blanc, 2007). Once inside the plant cell cytoplasm, viruses are uncoated and replicated following features similar to those described for animal viruses. In the second half of the 20th century, a number of studies have been conducted to uncover the mechanism(s) of virus entry into the plant cells (Shaw, 1985). Efforts made to investigate whether viruses enter plant cells via pinocytosis or attachment to specific cell-surface receptors following inoculation remained unfruitful. Observations of tobacco mosaic virus (TMV) and tobacco rattle virus (TRV) rod-shaped particles with their ends attached to the outer CW surface or to protoplasts after manual inoculation suggested that extracellular attachment site would facilitate cell entry of virion or RNA virus genome (Gaard and de Zoeten, 1979). However, virus attachment was not proved specific to susceptible hosts and no definitive evidence of virus entry upon attachment has been obtained so far. To the best actual knowledge, plant viruses cannot actively break the CW, and while endocytosis-like pathways have been observed in plants (Kitakura et al., 2011), viruses can neither use the endocytic pathway to enter cells surrounded by CWs nor exit them by budding. The absence of a lipoprotein envelope in most plant viruses probably represents an adaptation to the evolution of the CW in contrast to the enveloped viruses entering animal cells without CWs. In the few enveloped plant virus genera, i.e., *Tospovirus, Cytorhabdovirus, Nucleorhabdovirus*, and *Emaravirus*, the envelope facilitates vector-mediated virion transmission but is not required for cell entry and intercellular movement (Adkins, 2000; Jackson et al., 2005; Albornoz et al., 2016). Interestingly, the CW of *Chlorella* spp., a single-cell green algae sharing similar cell architecture with higher plants, can be actively penetrated by paramecium bursaria chlorella virus (PBCV-1). After enzymatic digestion of the CW, PBCV-1 gets fused to the cell membrane via the lipid bilayer membrane underneath the outer glycoprotein capsid and translocates its genome in the algae host (Van Etten, 2003).

## Virus Cell-To-Cell Movement Through the ECM

**FIGURE 2:**
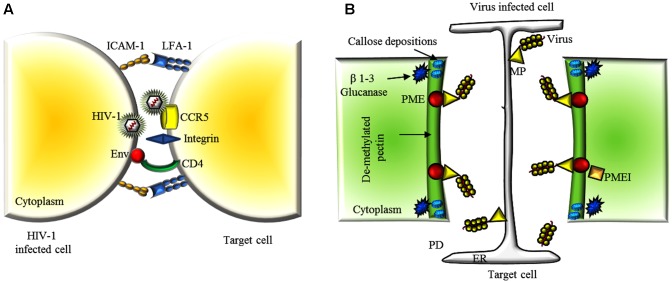
Extracellular matrix and CW dynamics in the viral cell-to-cell movement. **(A)** A schematic representation of a viral synapse between a HIV-1 infected T cell and a receptor-expressing target cell. HIV-1 envelope at the surface of the infected cell binds to α4β7 on the target cell. HIV-1 envelope glycoproteins (Env) are expressed on the infected cell PM and interact with the receptors CD4 and CCR5 or CXCR4 on the target cell. The adhesion molecules, intercellular adhesion molecule 1 (ICAM1) and lymphocyte function-associated antigen 1 (LFA1), engage integrin to stabilize the cellular conjugate. **(B)** Schematic representation of the concerted action of some endogenous and exogenous factors facilitating virus movement throughout PD. After viral penetration, plants reduce size exclusion limit of PDs by locally depositing callose at the neck regions. Virus infection triggers the activity of β 1-3 glucanases that facilitate gating of the PD pore in cooperation with PME and MP. Overexpression of PMEIs or silencing of PME delays virus cell-to-cell spreading by counteracting these processes and limiting PME/MP-mediated PD pore dilatation. PM, plasma membrane; ER, endoplasmic reticulum; CW, cell wall; PD, plasmodesmata; CP, coat protein; MP, movement protein; TMV, tobacco mosaic virus.

A successful viral infection relies on the ability of viruses to overcome multiple barriers and move from cell to cell (Zhong et al., 2013). In animal systems, two main biological strategies are known for an efficient virus cell-to-cell transmission. Viruses may exploit existing cell-cell interactions, such as neurological or immunological synapses or they may establish cell-cell contacts between cells that are not normally in physical contact (**Figure 2A**). The ability to utilize and manipulate cell-cell contact contributes to the success of viral infections. Many viruses including HSV, HIV and human T-lymphotropic virus (HTLV) can form so-called virological synapses: virus-induced specialized areas of contact between cells that promote cell-to-cell transmission (Vasiliver-Shamis et al., 2008; Abaitua et al., 2013). Virus infections can upregulate endogenous cell adhesion molecules (CAM), such as the protein ICAM-1, as well as other components of the extracellular matrix (Nakachi et al., 2011; Gross and Thoma-Kress, 2016). Some viruses can also produce their own adhesion proteins. Different viruses express the glycoprotein Env that can act as a viral adhesion molecule (VAM), mimicking the behavior of a CAM (Mothes et al., 2010).

Because a plant virus can move through the host via the symplast (Kumar et al., 2015), the entry process is completed when the virus has entered and infected the first cell, and breaching of surface layers of leaves and CW is no longer required. Nevertheless, the plant ECM plays a critical role also in traffic regulation in the symplasm. Plasmodesmata (PD), the intercellular organelles connecting the symplastic space between individual plant cells (Brunkard and Zambryski, 2017) are bordered by the PM and surrounded by CW conferring rigidity and shape to the organelle. Around PDs, the plant ECM is organized in micro-domains with specific composition and metabolism, partially yet unknown (Knox and Benitez-Alfonso, 2014). Plant viruses move through PD connections either as entire virions or ribonucleoprotein complexes. In either case, they encode one or more movement protein (MPs) allowing PD gating via molecular mechanisms not yet fully understood, and with the help of the host cytoskeleton and/or endomembranes that facilitate virus movement throughout the symplastic connections (Lucas, 2006; Harries et al., 2010; Harries and Ding, 2011).

## ECM and CW Remodeling During Viral Infection

Different evidence highlight the importance of the ECM in mediating responses to biotic stresses. ECM is a highly dynamic structure that continuously undergoes controlled remodeling. HCV infection in native liver and its recurrence post-transplant have been shown to significantly affect the deposition and remodeling of extracellular matrix (ECM) components, particularly collagen, leading to enhanced fibrosis (Borg et al., 2011). ECM remodeling is often mediated by the activity of specific degradative enzymes. MMPs-mediated remodeling is fundamental for maintenance of the ventricular structure and function during myocarditis, an inflammation of the myocardium associated with necrosis or degeneration of cardiomyocytes caused by many viruses such as enteroviruses, parvovirus B19, adenovirus and HCV. MMPs, also named matrixins, are calcium-dependent zinc-containing endopeptidases, able to degrade ECM proteins and to process bioactive molecules during pathological conditions, such as inflammation and tissue injury following inflammatory signals. They can mediate changes in ECM and affect immune and pro-inflammatory cell behavior (Liu et al., 2006; Wells et al., 2015; Peeters et al., 2017). MMP affect also disease severity in infants with respiratory syncytial virus (RSV) infection. MMP and their inhibitors contribute to the balance between ECM degradation and deposition, coordinating tissue healing (Schuurhof et al., 2012). Immune responses occur in the context of integrin-mediated adhesive interactions with the ECM. For example, during influenza infection, the α1β1 integrin, which binds collagen I and IV, mediates the retention of memory T cells in the lung after viral clearance, which is important for secondary immunity (Ray et al., 2004).

The plant CW also undergoes specific remodeling events during virus interaction. Callose (β -1,3-glucan), a polysaccharide synthesized in the CW by callose synthases and degraded by β-1,3-glucanases (Zavaliev et al., 2011), accumulates around the PD neck as a collar and its turnover controls PD transport capacity (Fitzgibbon et al., 2010; Guseman et al., 2010; Vaten et al., 2011; Han et al., 2014). The plant ECM can counteract the PD-gating function of viral MPs via localized apoplastic accumulation of callose around PD neck, and a fast and effectively reduction of the PD size exclusion limit (De Storme and Geelen, 2014). Evidence, such as the interaction of TMV MP with the ankyrin repeat-containing protein ANK and of potato virus *X* TGB2 with proteins associated with β-1,3-glucanase, suggests that some viral MPs have developed a counter-counter-reaction strategy to decrease callose accumulation and gate PD for cell-to-cell movement (Fridborg et al., 2003; Ueki et al., 2010; Zavaliev et al., 2011). More generally, demonstration that success of virus infection and callose accumulation around PD are inversely correlated (Iglesias and Meins, 2000; Zavaliev et al., 2013) indicates that callose accumulation at PD is an early barrier the ECM activates to block virus entry into neighboring cells and spread in the host (Epel, 2009).

Besides callose accumulation and cellulose content reduction, pectin composition around PD is also different from other CW regions. The specific composition of the complex group of pectic polysaccharides, prevalently low-methylesterified homogalacturonans (HG), found in the pectic microdomain at PDs, can influences CW porosity and rigidity among other factors (Orfila and Knox, 2000; Burton et al., 2010). Pectin Methyl Esterase (PME) and pectinase are found around PDs and are involved in HG de-methylesterification (Morvan et al., 1998; Pelloux et al., 2007). The activity of PME is regulated by pH, ionic strength and by PME inhibitors (PMEI) (Balestrieri et al., 1990; Di Matteo et al., 2005), which in turn can also modulate localized loosening of the CW and PD gating (Micheli, 2001; Pelloux et al., 2007; Peaucelle et al., 2011; Chebli and Geitmann, 2017).

Interestingly, PME interacts with the MP of TMV, turnip vein clearing virus (TVCV), cauliflower mosaic virus, and chinese wheat mosaic virus at the CW, and this interaction is essential for cell-to-cell movement of TMV (Chen et al., 2000; Andika et al., 2013). Furthermore, PME silencing or overexpression of PMEIs in *Nicotiana* spp. delay TVCV and TMV systemic movement and significantly reduce plant susceptibility to virus infection (**Figure 2B**) (Chen and Citovsky, 2003; Lionetti et al., 2014b; Bubici et al., 2015). While the mechanisms by which PMEs facilitate viral spread are yet unknown, the relative levels and timing of accumulation of PME and MP at PDs can have different effects on the permeability of the CW barrier and in turn on development of virus infection (Bubici et al., 2015; Lionetti et al., 2015).

## Biotechnological Applications: ECM in Oncolytic Virotherapy and Improvement of Plant Virus Resistance

The possibility to revert a tumor immunosuppression by virotherapy represents an interesting strategy to fight tumors. A key limitation is the targeting of the virus to the tumor. The ECM not only precludes virus spread but also virus arrival to the tumor. Therefore, arming the viruses with ECM-degrading enzymes and extending virus permissiveness to non-tumor stromal cells is currently actively explored to improve virus spread and virus constant targeting. Newcastle disease virus (NDV) is an avian paramyxovirus with a selective oncolytic effect on tumor cells in culture and in animal models (Matveeva et al., 2015). ECM limits spread of NDV and other viruses but the removal of tissue collagen and heparan sulfate by means of treatments with collagenase and heparinase before infection increases viral dissemination (Yaacov et al., 2012). Collagen, HA and HS also interfere with the oncolytic activity of adenovirus and HSV-1 (McKee et al., 2006; Guedan et al., 2010; Watanabe, 2010).

A deeper understanding of the diverse biological activities and properties of the plant CW must be attained to uncover the many parallel approaches that viruses use to overcome such barrier and eventually design innovative strategies for plant defense. Engineering a plant CW more resistant to virus vector insects, might be one possibility. In fact, aphid salivary secretions contain some CW degrading enzymes, such as polygalacturonases and pectin methylesterases that the insect use for stylet penetration (Dreyer and Campbell, 1984). A CW more resistant to enzymatic degradation could reduce viral transmission. The evidence that PMEIs limit viral spread suggests that this class of inhibitor may also be utilized in breeding programs aimed to obtain plant varieties less susceptible to virus diseases. Interestingly, the pectic oligogalacturonides and HA fragments can be perceived as damage-associated molecular patterns (DAMP), upon tissue injury or pathogen infection, activating the innate plant and animal immune system, respectively (Ferrari et al., 2013; Kavasi et al., 2017). The potential role of these fragments in response to virus infection and relative biotechnological application should be explored.

## Conclusion

In both plant and animal system, the ECM is of fundamental importance for regulation of active and reciprocal exchange of information between cells. Characteristics and properties of ECMs are also critical for viral entry, transmission and exit, and their differences in plant and animal cells have probably influenced the evolution of structural and functional properties of animal and plant viruses. The envelope surrounding animal viruses helps avoiding the host immune system and crossing the PM barrier in both directions via endo- and exocytosis, respectively. Plant viruses, coping with a CW that cannot be actively penetrated, cannot make the same use of envelopes but developed expression of unique MPs that facilitate cell-to-cell movement within a host. Despite the differences highlighted above, plant and animal ECMs share many of their compounds, e.g., uronic acid that is a main constituent of the CW pectic matrix as well as the GAGs receptor in the animal ECM. It is striking to observe that in both matrixes viruses can exploit these compounds to enter the host cell, albeit with different strategies. Future efforts are needed to understand the role of specific plant CW polysaccharides around PD in viral cell to cell movement as well as to elucidate possible roles of CW in virus entry. This knowledge could provide new targets for the genetic improvement of plant resistance to viruses.

The role of the ECM in cancer is of particular interest as a significant contributor to tumor progression. Furthermore, the evidence that the matrisome and key ECM remodeling effects can influence certain diseases offers new perspectives for therapeutic intervention. Current clinical trials using inhibitors of ECM-related targets are ongoing and promising. As the ECM is actively remodeled, targeting specific individual ECM components as well as timing the therapy correctly deserve an intense focus in future research to uncover new targets for future therapy.

## Author Contributions

LS and VL equally participated in drafting, writing and revising the article. VL prepared the figures.

## Conflict of Interest Statement

The authors declare that the research was conducted in the absence of any commercial or financial relationships that could be construed as a potential conflict of interest.
